# The gram-negative sensing receptor PGRP-LC contributes to grooming induction in *Drosophila*

**DOI:** 10.1371/journal.pone.0185370

**Published:** 2017-11-09

**Authors:** Aya Yanagawa, Claudine Neyen, Bruno Lemaitre, Frédéric Marion-Poll

**Affiliations:** 1 Research Institute for Sustainable Humanosphere, Kyoto University, Uji, Japan; 2 Global Health Institute, School of Life Sciences, Swiss Federal Institute of Technology, Lausanne, Switzerland; 3 Evolution, Génomes, Comportement & Ecologie, CNRS, IRD, Université Paris-Sud, Université Paris-Saclay, Gif-sur-Yvette, France; 4 AgroParisTech, Département Sciences de la Vie et Santé, Paris, France; Biomedical Sciences Research Center Alexander Fleming, GREECE

## Abstract

Behavioral resistance protects insects from microbial infection. However, signals inducing insect hygiene behavior are still relatively unexplored. Our previous study demonstrated that olfactory signals from microbes enhance insect hygiene behavior, and gustatory signals even induce the behavior. In this paper, we postulated a cross-talk between behavioral resistance and innate immunity. To examine this hypothesis, we employed a previously validated behavioral test to examine the function of taste signals in inducing a grooming reflex in decapitated flies. Microbes, which activate different pattern recognition systems upstream of immune pathways, were applied to see if there was any correlation between microbial perception and grooming reflex. To narrow down candidate elicitors, the grooming induction tests were conducted with highly purified bacterial components. Lastly, the role of DAP-type peptidoglycan in grooming induction was confirmed. Our results demonstrate that cleaning behavior can be triggered through recognition of DAP-type PGN by its receptor PGRP-LC.

## Introduction

Insect defenses against pathogens have been mostly studied from an immunological point of view, unravelling the biochemical components as well as the signaling pathways involved in triggering these responses [1]. However, little data is available concerning the role of behavior in these innate defenses. Although hygienic activities like grooming (have been shown recently to be) are remarkably developed in social insects [2–4], little is known about grooming reflexes in *Drosophila*. *Drosophila* clean themselves vigorously (*i*.*e*. grooming) when touched with bacterial extracts, or with bitter chemicals (like quinine), but not with sugar or water [5]. Our objective was to study self-grooming in a solitary insect amenable to genetic studies. We used *Drosophila melanogaster* to establish whether grooming behavior relies on the immune system, and if so, which chemical signals from microbes (bacteria, fungi) might stimulate self-grooming.

In *Drosophila*, antimicrobial defense mechanisms include maintenance of physical barriers (epithelia), secretion of humoral mediators (antimicrobial peptides, reactive oxygen species), activation of proteolytic cascades leading to melanisation and cellular functions including phagocytosis and encapsulation [1]. The regulation of the antimicrobial peptide gene expression during systemic infection has been studied in great detail. Production of antimicrobial peptides by the fat body, an analogue of the mammalian liver, is orchestrated through two signaling modules: the Toll and Imd pathways are activated in response to microbial infection and lead to activation of NF-κB-like factors. Two types of pattern recognition systems assure microbial sensing upstream of the Toll and Imd pathways. Peptidoglycan recognition proteins (PGRPs), which can be soluble or membrane-bound, activate both Toll and Imd, and soluble Glucan-binding proteins (GNBPs), which recognize yeast glucans and activate exclusively Toll pathways. All PGRPs recognize bacterial peptidoglycan (PGN) but with specificity towards the nature of peptidoglycan: Lysine-type PGN from Gram-positive bacteria engages the soluble form PGRP-SA in hemolymph and leads to Toll activation, while the Imd pathway, active against Gram-negative bacteria, is triggered by the binding of DAP-type PGN to PGRP-LC in cooperation with PGRP-LE [6–8]. The Imd pathway mainly responds to Gram-negative bacterial infection and controls antibacterial peptide genes via the activation of the Rel protein Relish. PGRP-LB is an amidase specific of DAP-type PGN and its expression is controlled by the Imd pathway [9, 10]. PGRP-LB negatively regulates the Imd pathway by scavenging extracellular peptidoglycan. PGRP-LC is a transmembrane receptor with three alternative splice isoforms (PGRP-LCa, -LCx and -LCy) that have somewhat different ligand specificities. Antimicrobial peptides are also expressed in many epithelia in contact with the external world such as the gut, genital tracts and trachea. Tissue-specific expression of surface and intracellular receptors ensures a correct level of immune activation in tissues exposed to the environment versus tissues in contact with the sterile interior milieu [11, 12].

**Fig 1:**
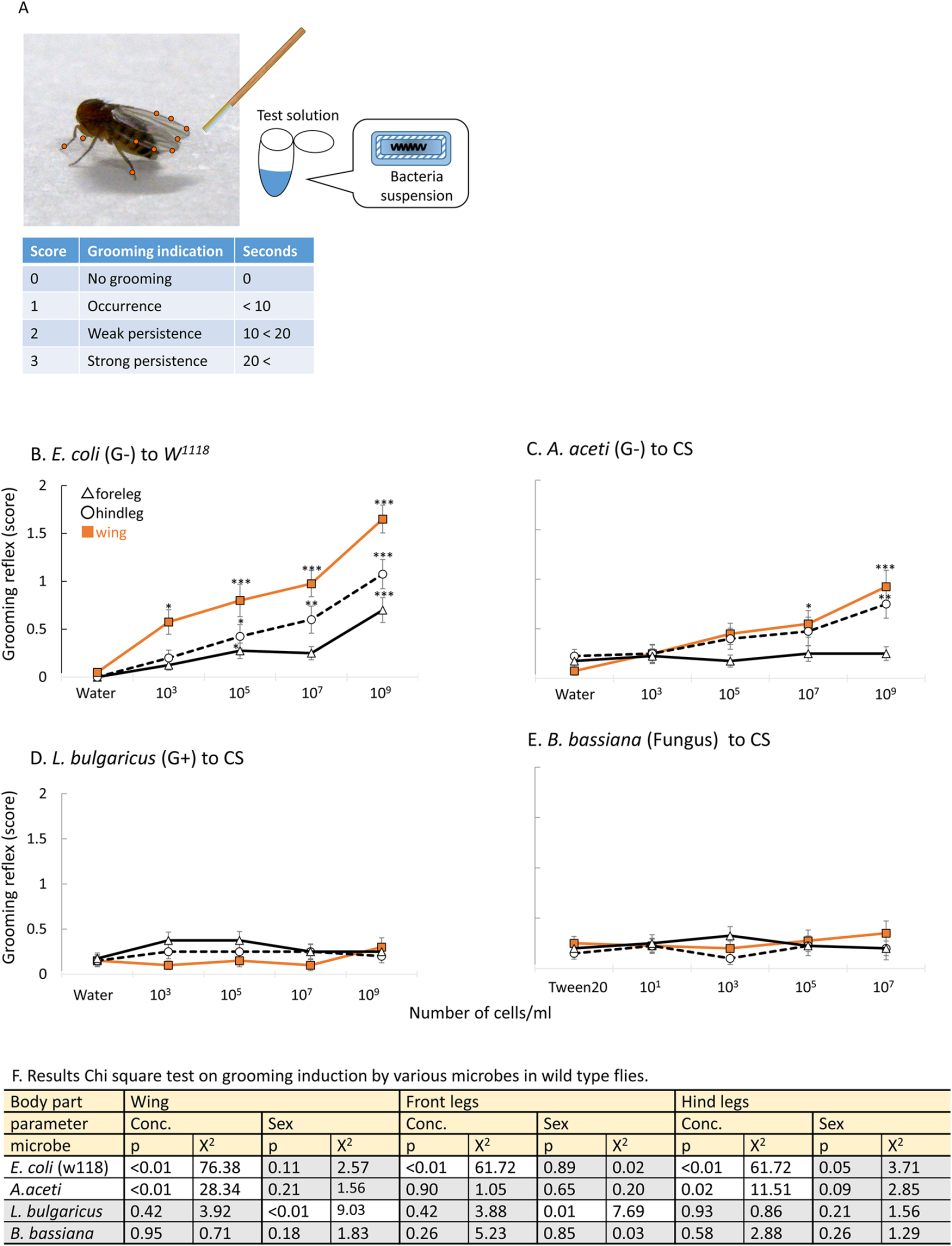
(A) Grooming induction test. Circles on body parts indicate location of gustatory receptors. Tested locations of gustatory receptor are indicated by red circle (front legs, hind legs and wing). (B)–(E) show the results. Grooming behavior induced by (B) heat-killed *E*. *coli* on *w*^*1118*^, (C) *A*. *aceti* on CS flies, (D) *L*. *bulgaricus* on CS flies and (E) *B*. *bassiana* on CS flies. n = 40 (n = 20 from each sex). G-/+ means Gram-negative/positive bacteria. Significant increase from the control response (water) is indicated by asterisks: * indicates p <0.05, **indicates p < 0.01 and *** indicates p < 0.001 (Dunnett’s test). Data represent mean ± standard error (SE). (F) shows results of Chi-square test on grooming induction by various microbes in wild type flies. Microbes, which induced significant concentration-dependent behavioral increase were in white zone, and no-significance in gray zone. White zone in a table shows significant difference (p < 0.05), and grey zone indicates no-significance (p > 0.05).

Here we postulated that microbial factors, notably peptidoglycan from Gram-negative bacteria, plays an important role in inducing hygiene behavior in *Drosophila*. Grooming behavior in *Drosophila* may contribute to diminishing the exposure of flies to pathogens. Previous studies have shown that a stereotyped grooming reflex can be triggered in decapitated flies by tactile [13, 14] or chemical stimulation [5, 15, 16]. As a consequence, use of decapitated flies enable simple but reliable assessments of grooming activity upon stimulation of sensilla [17, 18]. We have carefully modified the method to remove the influence from mechanical stimulus together with water influence and confirmed it with several approaches using UAS-GAL4 experiments and optogenetic experiments[5], then applied this classical method to assess the reflex triggered by chemical stimuli associated with pathogens. We scored the occurrence of grooming responses following a contact with a solution of water mixed with different solutions. First, microbes, which activate different pattern recognition systems, were applied to decapitated flies to assess the function of bacterial components in the induction of grooming. Then the grooming induction tests were conducted with highly purified bacterial components to pinpoint specific elicitors. These tests support an important role of DAP-type PGN in eliciting grooming. Lastly we showed that two pattern-recognition receptor capable of recognizing DAP-type PGN working upstream of the Imd pathway are required for grooming. In contrast, PGRP-LB and intracellular components of the Imd pathway, Relish, and the Imd adaptor [19] were not involved in the grooming reaction (Fig 1A). In this paper, we successfully demonstrate that DAP-type PGN from Gram-negative bacteria triggers the cleaning behavior when it is recognized by its receptor PGRP-LC.

## Results

### Contact with microbes induces grooming

To determine whether microbial compounds elicit grooming behavior in *Drosophila*, grooming activity in decapitated flies was examined after applying different microbial species. Gram-negative bacteria, gram-positive bacteria, and pathogenic fungi were tested.

Grooming induction by *E*. *coli*, a representative Gram-negative bacterium, was already shown in Canton S (CS) flies [5] (Figure a in S1 Fig). Since it cannot be excluded that the grooming behavior is highly dependent on the genetic background of the flies, we tried to reproduce *E*. *coli*–induced grooming in another wild-type strain, *w*^*1118*^. *E*. *coli* suspension successfully induced the behavior in *w*^*1118*^ in a dose dependent manner (Fig 1B). In addition, we employed another common Gram-negative bacterium, *Acetobacter aceti*, to confirm that Gram-negative bacterial components induced the cleaning behavior in flies. *A*. *aceti*, exhibited a concentration dependent behavioral induction upon contact with hind legs (p = 0.02, x^2^ = 11.51, Chi-square test, Fig 1F) and wing margins (p < 0.01, x^2^ = 28.34, Chi-square test, Fig 1F), but not with front legs (p = 0.90, x^2^ = 1.05, Chi-square test, Fig 1F) (Fig 1C). There was no difference in behavioral induction between females and males both in CS and W^1118^ flies to Gram-negative bacteria (front legs: p > 0.10, hind legs: p > 0.05, wing margins: p > 0.10, Chi-square test, Fig 1F), so we pooled male and female responses in all further experiments.

On the other hand, no grooming induction was observed upon contact with Gram-positive bacteria, *Lactobacillus bulgaricus* and *Mycoplasma fermentans* (Fig 1D, Figure b in S1 Fig, respectively) and fungi, *Beauveria bassiana* (Fig 1E) (p > 0.1 for all body parts regardless of concentration and sex parameters).

These results indicate that bacterial components from Gram-negative bacteria contribute to inducing grooming behavior in decapitated flies.

### Highly purified microbial components induce grooming

To determine which molecules from Gram-negative bacteria are detected by *Drosophila*, grooming activity in decapitated flies was examined using a standard solution of LPS from SIGMA containing also bacterial peptidoglycan and lipopeptide (referred to as Sigma ‘LPS’)[20] and highly purified microbial elicitors, including Lipopolysaccharide (LPS), two preparations of peptidoglycans and glucan.

**Fig 2:**
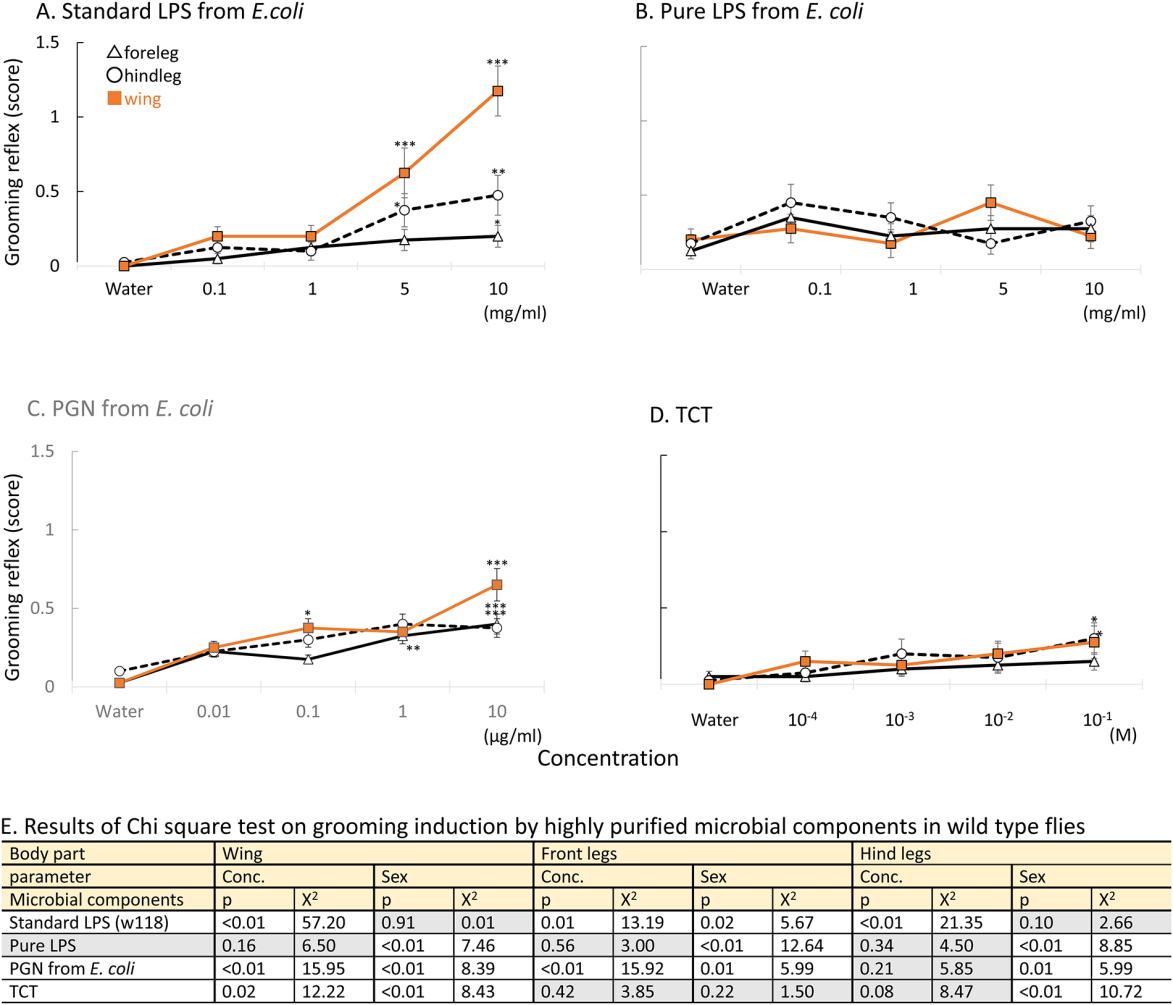
Grooming behavior induced by microbial components in wild-type flies, (a) standard LPS from E. coli on *w^1118^* flies (b) pure LPS from E.coli on CS flies, (C) peptidoglycan from E. coli, (D) TCT on CS flies. High grooming response was induced by Standard LPS from E. coli on CS flies. The data was available in Yanagawa et al. [5] A significant increase in response from that of the control (water) is indicated by asterisks: * indicates p < 0.05, ** indicates p < 0.01, and *** indicates p < 0.001 (Dunnett’s test). (E) shows results of Chi-square test on grooming induction by highly purified microbial components in wild type flies. Compounds, which induced significant concentration-dependent behavioral increase were in white zone, and no-significance in gray zone. White zone in a table shows significant difference (p < 0.05), and grey zone indicates no-significance (p > 0.05).

Although we had previously seen a clear grooming induction by a standard solution of Sigma ‘LPS’ from *E*. *coli* (L2630, Sigma) (Fig 2A) [5], this behavioral induction disappeared with a highly purified solution of LPS from *E*. *coli* (tlrl-eblps, InvivoGen) (p > 0.1 for all body parts regardless of concentration) (Fig 2B). In contrast, peptidoglycan from *E*. *coli* (gift from Dominique Mengin-Lecreulx) induced the cleaning behavior significantly (p < 0.01, x^2^ = 15.95 at concentration parameter, p < 0.01, x^2^ = 0.39 at sex parameter, Chi-square test) (Fig 2C). Grooming induction was also observed after stimulation with tracheal cytotoxin (TCT, gift from Dominique Mengin-Lecreulx) (p = 0.02, x^2^ = 12.22 at concentration parameter, p < 0.01, x^2^ = 8.43 at sex parameter, Chi-square test) (Fig 2D). TCT is a soluble fragment of peptidoglycan composed of a monomer with an anhydro bound released from the cell wall of proliferating Gram-negative bacteria [21]. It has been shown to be the minimal peptidoglycan unit capable of activating an immune response [20, 22]. However, peptidoglycan from the DAP-type Gram-positive bacterium, *Bacillus subtilis* (tlrl-pgnb3, InvivoGen) induced grooming less conclusively (Figure c in S1 Fig: p > 0.1 for all body parts at concentration parameter) and the soluble ß-glucan laminarin (tlrl-lam, InvivoGen) did not induce the behavior (Figure d in S1 Fig: p > 0.1 for all body parts at concentration parameter). Results of Chi-square test are listed in Fig 2E. Of note, a low behavioral induction was observed in the comparison between control and peptidoglycan of *B*. *subtilis* when it was deposited on hind legs, but only at the highest concentration (Figure c in S1 Fig, Dunnett’s test). The results of Chi-square test are listed in Fig 2E and S1 Table.

Taken together, these results suggest that peptidoglycan from Gram-negative bacteria plays a role in inducing the grooming reflex. In all following experiments, we kept using the standard LPS from Sigma because it is a cheap and convenient source of peptidoglycan.

### Grooming induction is lost in mutants of pattern-recognition receptors but not of signaling components of the Imd pathway

**Fig 3:**
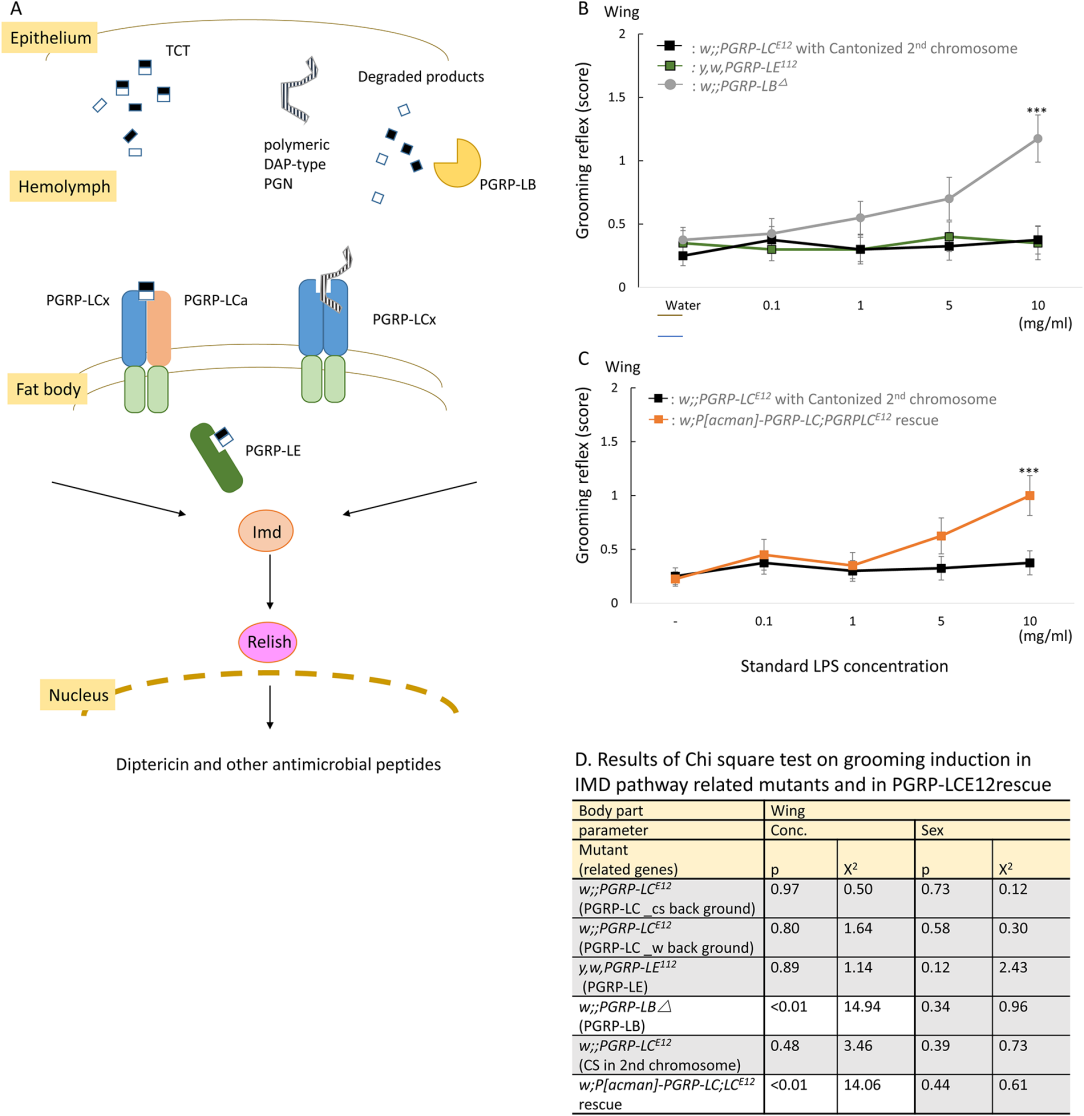
(A) Schematic indicating how the Imd pathway is activated by Gram-negative bacteria. Grooming behavior induced by LPS contact on wings in (B) black square: w;;*PGRP-LC^E12^* with Cantonized 2nd chromosome, green square: y,w,*PGRP-LE^112^* and grey circle: w;;PGRP-LBΔ (C) black square: w;;PGRP-LC^E12^ and orange square: w;P[acman]-*PGRP-LC;PGRPLC^E12^* rescue flies. Standard LPS was used as a stimulus. A significant increase in response from that of the control (water) is indicated by asterisks: * indicates p < 0.05, ** indicates p < 0.01, and *** indicates p < 0.001 (Dunnett’s test); n = 40 (n = 20 for each sex). Data represents mean +/- SE, analyzed as in Fig 1. (D) shows results of Chi-square test on grooming induction. Mutants, which showed significant concentration-dependent behavioral increase were in white zone, and no-significance in gray zone. White zone in a table shows significant difference (p < 0.05), and grey zone indicates no-significance (p > 0.05).

Since *Drosophila* senses Gram-negative bacterial peptidoglycan via two PGRPs, membrane-bound PGRP-LC and intracellular PGRP-LE [7, 8, 11, 12], we used mutants of these receptors (*w;;PGRP-LC*^*E12*^ and *y*, *w*, *PGRP-LE*^*112*^) to test their role in the activation of grooming by DAP-type PGN. Additionally, we tested a mutant of other Imd pathway components, namely the negative regulator PGRP-LB (*w;;PGRP-LB*^*Δ*^), a secreted amidase which binds to and degrades extracellular peptidoglycan (Fig 3A)[23, 24]. The results of Chi-square test are listed in Fig 3D and S2 Table.

**Fig 4:**
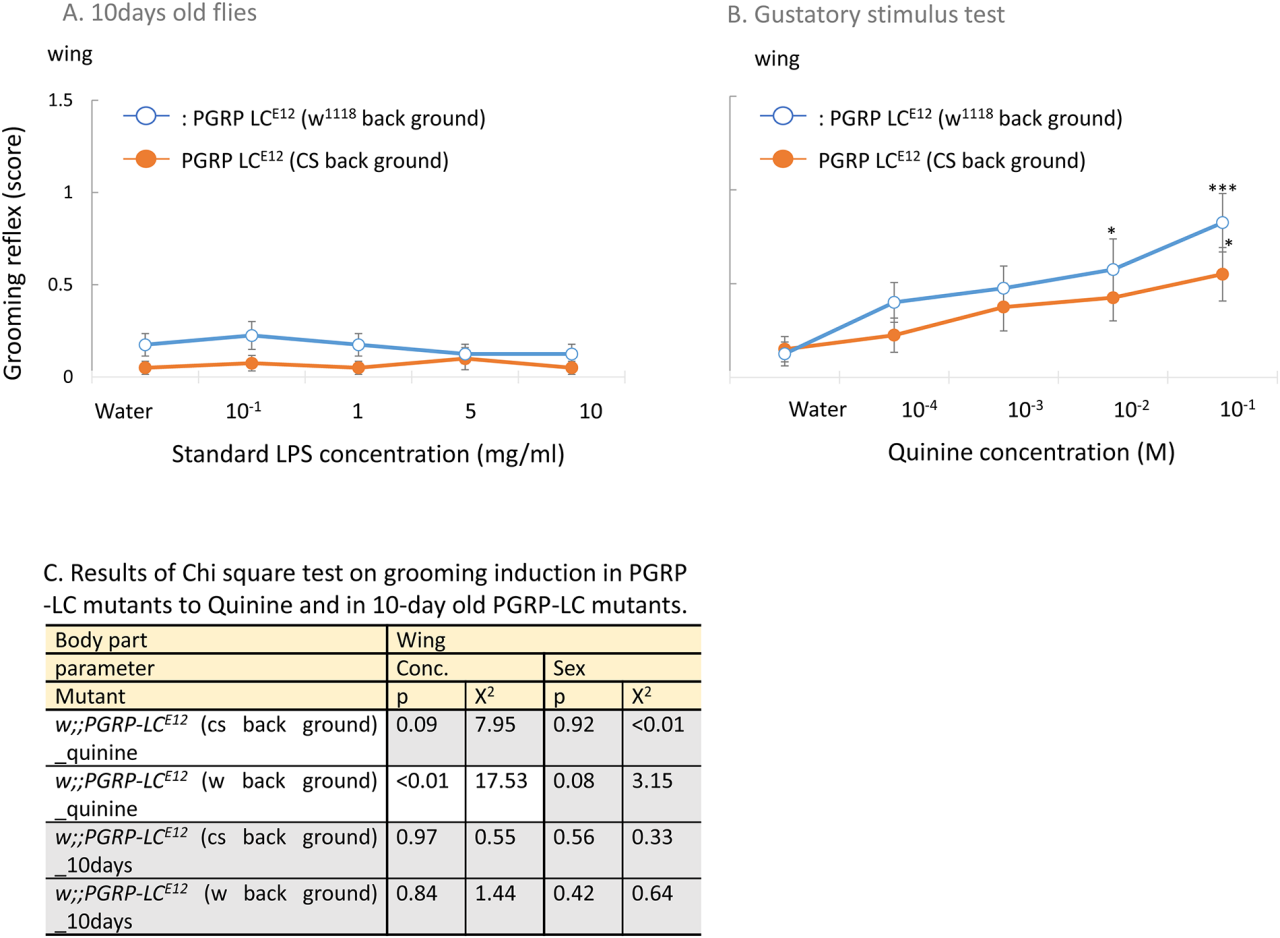
(A) Grooming behavior induced in 10-day old *PGRP-LC*^*E12*^ mutants in orange circle with orange line: CS background and white circle with blue line: *w*^*1118*^ background. (B) Grooming behavior induced by quinine in *PGRP-LC*^*E12*^ mutants of orange circle with orange line: CS background and white circle with blue line: *w*^*1118*^ background flies. A significant increase in response from that of the control (water) is indicated by asterisks: * indicates p < 0.05, ** indicates p < 0.01, and *** indicates p < 0.001 (Dunnett’s test). (C) shows the results of Chi-square test in PGRP-LC mutants to Quinine and in 10-day old PGRP-LC mutants. Mutants, which showed significant concentration-dependent behavioral increase were in white zone, and no-significance in gray zone. White zone in a table shows significant difference (p < 0.05), and grey zone indicates no-significance (p > 0.05).

Grooming induction was significantly suppressed in all mutants lacking PGRPs (either *PGRP-LC*s or *PGRP-LE*), indicating an involvement of these pattern-recognition receptors in the activation of grooming upon DAP-type PGN (Fig 3B, S2 Fig). In this study, we confirm PGRP-LC function in grooming induction both in 4-day old flies and 10 day-old flies (Fig 4A and Figure a in S3 Fig) since, in previous results, age dependent increase was observed in grooming induction [5]. To control for potential background effects, we first used several strains deleted for *PGRP-LC* in different wild-type backgrounds (*w*^*1118*^, *Can*^*S*^) and obtained similar results. The response was rescued by the reinsertion of the *PGRP-LC* locus into the *PGRP-LC*^*E12*^ mutant background (full genotype: *w;P[acman]-PGRP-LC;PGRP-LC*^*E12*^) (Fig 3C). There was no significant difference in the response in mutants *w;;PGRP-LC*^*E12*^ and *y*, *w*, *PGRP-LE*^*112*^ (p = 0.41, x^2^ = 0.68 at strain parameter, Chi-square test), while *w;;PGRP-LB*^*Δ*^ showed drastic behavioral increase. Grooming was still observed in *PGRP-LB* deficient flies at normal levels, suggesting that PGRP-LB is not involved in this process. Moreover *w;P[acman]-PGRP-LC;PGRPLCE12* rescue clearly recovered the response in comparison with *w;;PGRP-LC* mutants (p < 0.01, x^2^ = 162.43 at strain parameter, Chi-square test)(Fig 3C). We also checked whether grooming induction by gustatory stimulus (quinine, bitter taste) was dependent on the same receptor, PGRP-LC. However, it seemed to be independent (Fig 4B, Figure b in S3 Fig). The results of Chi-square test are listed in Fig 4C and S3 Table.

**Fig 5:**
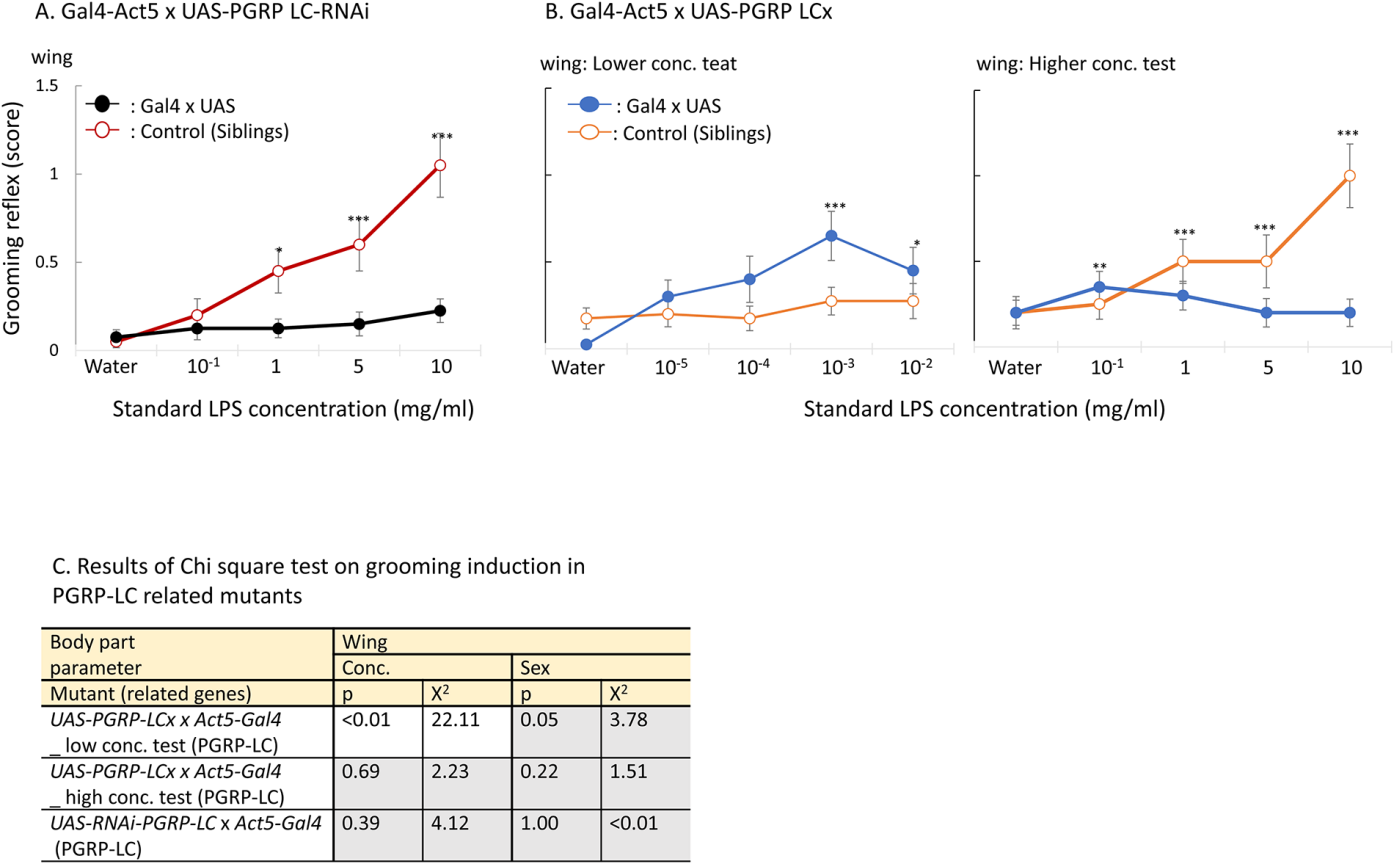
(A) Grooming behavior induced in offspring of act-Gal4 x PGRP-LC^RNAi^ flies black circle with black line: *actGal4x -PGRP-LC^RNAi^* and white circle with red line control (siblings). Standard LPS was used as a stimulus. (B) Grooming behavior induced by LPS contact on wing and front/hind legs in PGRP-LCx overexpressing flies by Gal4-UAS system. Concentration dependent 40 repetitions were conducted by two concentration range: 10^−5^–10^−2^ mg/ml range of standard LPS and 10^−1^–10 mg/ml range of standard LPS. Open circles illustrated the grooming induction in control flies (siblings). Standard LPS was used as a stimulus. A significant increase in response from that of the control (water) is indicated by asterisks: * indicates p < 0.05, ** indicates p < 0.01, and *** indicates p < 0.001 (Dunnett’s test). n = 40 (n = 20 for each sex). Data represents mean +/- SE, analysed as in Fig 1. (C) shows the results of Chi-square test. Mutants, which showed significant concentration-dependent behavioral increase were in white zone, and no-significance in gray zone. White zone in a table shows significant difference, and grey zone indicates no-significance.

Next, we performed ubiquitous mis-expression of PGRP-LCx using the Gal4-UAS system and RNAi/overexpression constructs. Flies expressing *RNAi* against *PGRP-LC* did not show the response (Fig 5A, Figure a in S4 Fig), in agreement with what was observed in *PGRP-LC* mutants. When *PGRP-LCx* was overexpressed, flies became highly sensitive and induced behavior at 1000 times lower peptidoglycan concentration (Fig 5B, Figure b in S4 Fig). Then the grooming reflex had diminished. This pattern of output decrease appears sometimes also in other insect behaviors. It is considered as the neural adjustment cause by too strong stimulus, which caused bursts of electrophysiological signals in the neural circuit or by the systematic inhibition to regulate a reaction. The results of Chi-square test are listed in Fig 5C and S4 Table.

Taken together, these observations suggest that sensing of peptidoglycan from Gram-negative bacteria by PGRPs induces cleaning behavior. All results suggested a role of PGRP-LC in the induction of grooming behavior.

## Discussions

We examined the reflex of hygiene behavior of flies to microbial contact. Bacterial cell wall components were applied to decapitated flies to see the behavioral reflex to soluble bacterial compounds^5^. *Drosophila* senses Gram-negative bacteria via PGRP-LC and PGRP-LE and relays sensing of extracellular or intracellular DAP-type PGN to the Imd pathway [7, 25]. This study demonstrates that initial sensing of Gram-negative bacteria at epithelial surfaces may serve to resist microbial infection by inducing grooming behavior.

It is often said that insects combine behavioral resistance and immune activity [4, 26]. Since behavior likely has the lowest cost to prevent pathogenic infection for insects, they might use behavior as the first protection together with the cuticle barrier, and spend less resource on immune responses in the gut or the haemolymph, which might be a good trade-off system to save bio-energy [26]. We have reported that contacting wing marginal sensilla with *E*. *coli* and standard LPS solution induced grooming activity [5], which suggested that hygiene behavior in *Drosophila* can be triggered by a detection of microbial surface compounds by similar mechanisms/receptors to those involved in triggering immune responses. Using highly purified bacterial cell wall components, we could show that grooming can be induced upon deposition of peptidoglycan extracts derived from gram-negative bacteria. Peptidoglycan is recognized by PGRPs, which activate the Imd pathway [27, 28]. In this study, using the same methods that we applied to see the role of gustatory perception in grooming induction reflex in decapitated flies, we showed that the innate immune system also serves in behavioral resistance at surface level in *D*. *melanogaster*. The results show that classical components involved in bacterial recognition can be re-used to induce behavioral response.

Insect perception of microbes is still ambiguous. As for relations between the immune and the sensory systems, several transcriptomic studies noted that microbial infections are associated with changes of expression of olfactory-related genes like odorant binding proteins [29, 30]. Previously, we demonstrated that grooming in response to microbes relies in part on the activation of taste neurons because *poxn70* did not show any cleaning behavior by touching with bacterial extracts [5]. *Poxn* mutation in *poxn* flies induces a failure in the development of external chemoreceptors [31], and therefore, they are tasteless. Interestingly, we now show that grooming induction by microbes seems to depend on pattern-recognition receptors involved in immunity, since PGRP-LC and PGRP-LE mutants stopped responding to peptidoglycan. Whether PGRP-LC is expressed in taste neurons is presently unknown. To learn PGRP expression and interactions in the hemolymph of sensory sensilla, further studies are needed. It suggests that the surface immune-components serve in behavioral resistance. This is probably to prevent the loss of bioenergy because behavioral resistance works most effectively over the first contact with microbes. The interaction among signals and their cascades can much more complicated than we have thought. Even many other signals and its related genes can be involved in this behavior [16, 32] cooperating with local neurons [18]. Grooming induction by Sigma LPS was dramatic, while that by pure PGN was rather modest. In addition, sex dependent difference appeared more in behavioral responses induced by the compounds including those from gram-positive bacteria. It indicated that not only DAP-type PGNs but also the other bacterial surface compounds like lipopeptide would have some impacts on this behavioral reflex [5, 15, 32]. Moreover, although whether PGRP-LC is expressed in taste neurons is unknown, it is highly possible that standard LPS contains some substances perceived as aversive stimulus by *Drosophila* since the ion channel, which relates with aversive gustation, is known to have some interaction with standard LPS [33].

The role of grooming behavior seems diverse and many factors involved in this behavior are still unknown [32]. Grooming possibly helps removing dust particles [34] and also cleaning external chemosensory receptors [35]. Although self-grooming activities can be induced by a number of situations involving aversive stimuli, mechanical [36], chemicals [37] or complex behaviors like feeding and oviposition [38, 39], its importance for the survival of the species has rarely been considered. The hypotheses of grooming function to protect themselves against microbial infection were expressed independently in recent papers but not within a common unifying frame. In 2016, Soldano et al. [33] demonstrated that the role of ion channel dTRPA1 in the behavior after the chemical contact with Lipopolysaccharides (LPS), and in our knowledge, it is the first report of detailed mechanism on the link between behavioral immunity and insect perception. Vice versa, bacteria is known to use insect perception, and control its behavior to aid their survivals [40]. There is still a long way to go to recognize behavior as an integral part of the strategies used by insects to cope with pathogens [2, 3]. The behavioral process of grooming is highly complex and further study will open highly interesting aspects on the strategy for survival in insects which can bring new technique in the way of organic agriculture using biocontrol agents, insect mass production and sanitations in medical field.

## Materials and methods

### Fly stocks

*D*. *melanogaster* was maintained on a standard cornmeal agar food at 20°C and at 80% humidity. *CantonS* (*CS*) and white *w*^*1118*^ flies were used as wild-type controls. As for immunity mutants, *w;;PGRP-LC*^*E12*^ (in *w*^*1118*^ background or with Cantonized 2^nd^ chromosome) *y*, *w*, *PGRP-LE*^*112*^ and *w;;PGRP-LB*^*△*^ were used. *PGRP-LC*^*E12*^, *PGRP-LB*^*Δ*^and *PGRP-LE*^*112*^ lines have been described previously [6, 7, 9, 41–43]. 4 day old flies were used.

The Gal4-UAS system [44] was used to mis-express PGRP-LC. The *y*, *w;act5-Gal4* driver line (DGRC# 107727) was crossed to w;;UAS-PGRP-LCx or to w;;UAS- PGRP-LC^RNAi^. In Gal4-UAStests, balanced siblings were employed as controls.

### Microbial preparations

We tested the Gram-negative bacteria *Escherichia coli* and *Acetobacter aceti*, the Gram-positive bacteria *Lactobacillus bulgaricus*, *Listeria monocytogenes* and *Mycoplasma fermentans*, and the entomopathogenic fungus *Beauveria bassiana*.

The *E*. *coli* strain of TOP 10 was grown in liquid LB medium at 37°C. For heat-killed preparations, *E*. *coli* was washed with distilled water and heated at 95°C for 5 minutes. *A*. *aceti* ATCC 53264 was grown in liquid SH medium at 28°C. *A*. *aceti* was washed by distilled water and heated at 95°C for 5 minutes. 7.9 x 10^9^ /ml bacterial suspension was diluted 10^0^, 10^2^, 10^4^ and 10^6^ fold. *L*. *bulgaricus* was grown in liquid MRS medium at 40°C, then it was washed by distilled water and heated at 95°C for 5 minutes.

Heat-killed *Listeria monocytogenes* and *Mycoplasma fermentans* were purchased from InvivoGen, (InvivoGen, Lot # HKLM-35-03 and HKMF-32-01, respectively) and adjusted to 1.0 x 10^9^ / ml. The suspensions were diluted 10^0^, 10^2^, 10^4^ and 10^6^ fold. *B*. *bassiana* was grown in liquid LB medium for fungi. *B*. *bassiana* was washed by distilled water and heated at 95°C for 5 minutes. All microbial suspensions were adjusted to highest concentration, and then was diluted 10^0^, 10^2^, 10^4^ and 10^6^ fold. They have a pH of about 7.0.

### Chemicals

Standard LPS from *E*. *coli* was purchased from Sigma (L2630)[5], and highly purified LPS from *E*. *coli* was purchased from InvivoGen (tlrl-eblps). Peptidoglycan from the Gram-positive bacterium *Bacillus subtilis* and the soluble ß-glucan laminarin were purchased from InvivoGen (tlrl-pgnb3, tlrl-lam, respectively). Peptidoglycan from *E*. *coli* and TCT were gifts from Dominique Mengin-Lecreulx (University of Orsay, Orsay, France). All chemical suspensions have a pH of about 7.0.

### Grooming induction and scoring

Briefly, 10 flies were beheaded by a single cut made at the neck with micro-scissors. Micro-scissors were washed and wiped by 70% ethanol before and after use. Beheaded flies were placed in an upright position on a clean paper sheet and allowed to recover. To stimulate them, the wings, front legs, or hind legs were gently touched with a sharpened toothpick that was previously soaked in a test solution. To avoid contamination, the paper sheet was changed between each test, and a new toothpick was sharpened before each test. Grooming behavior after touching by toothpick was observed, and its intensity scored as 0, 1, 2, or 3. A score of 0 indicated no grooming, and a score of 1 or greater indicated that grooming occurred. Because grooming duration has been shown varied widely in previous studies, the strength of induction was scored as follows: 1, grooming that stopped within 10 seconds; 2, grooming that lasted more than 10 seconds and less than 20 seconds; and 3, grooming that lasted over 20 seconds. Four-day-old flies were used in testing. Each substance was tested on 20 females and 20 males (n = 40).

### Statistical analysis

To examine concentration-dependent increases in grooming behavior in headless flies with respect to sex, chemical, and fly strain, Chi-square test (JMP 10.0 software, SAS) was applied. Additionally, Dunnett’s test (JMP 10.0 software, SAS) was conducted to examine behavioral induction at each concentration.

## Supporting information

S1 Fig Grooming behavior induced by heat-killed microbes and its related chemicals in CS flies, (a) *E*.*coli* (b) *M*. *fermentans* (c) peptidoglycan from *B*. *subtilis* (d) ß-glucan from algae (laminarin) on CS flies were used for all tests, which employed *w*^*1118*^. A significant increase in response from that of the control (water) is indicated by asterisks: * indicates p < 0.05, ** indicates p < 0.01, and *** indicates p < 0.001 (Dunnett’s test). Please note that the data of CS flies to *E*. *coli* is provided as a reference. This data belongs to the previous publication in Front. Behav. Neurosci. [5].
(TIF)

S2 Fig Grooming behavior induced by LPS contact on front legs and hind legs in black square: *w;;PGRP-LC*^*E12*^ with Cantonized 2nd chromosome, green square: *y*, *w*, *PGRP-LE*^*112*^, grey circle: *w;;PGRP-LB*^*Δ*^, black square: *w;;PGRP-LC*^*E12*^ and orange square: *w;P[acman]-PGRP-LC;PGRP-LC*^*E12*^ rescue flies. Standard LPS was used as a stimulus. n = 40 (n = 20 for each sex). Data represents mean +/- SE, analyzed as in Fig 2. A significant increase in response from that of the control (water) is indicated by asterisks: * indicates p < 0.05, ** indicates p < 0.01, and *** indicates p < 0.001 (Dunnett’s test).
(TIF)

S3 Fig (a) Grooming behavior induced in 10-day old *PGRP-LC*^*E12*^ mutants in orange circle with orange line: CS background and white circle with blue line: *w*^*1118*^ background. (b) Grooming behavior induced by quinine in *PGRP-LC*^*E12*^ mutants of orange circle with orange line: CS background and white circle with blue line: *w*^*1118*^ background flies. Standard LPS was used as a stimulus. n = 40 (n = 20 for each sex). Data represents mean +/- SE, analysed as in Fig 2. A significant increase in response from that of the control (water) is indicated by asterisks: * indicates p < 0.05, ** indicates p < 0.01, and *** indicates p < 0.001 (Dunnett’s test).
(TIF)

S4 Fig (a) Grooming behavior induced in offspring of act-Gal4 x PGRP-LC^*RNAi*^ flies black circle with black line: actGal4x-PGRP-LC^*RNAi*^ and white circle with red line control (siblings). (b) Grooming behavior induced by LPS contact on front/hind legs in PGRP-LCx overexpressing flies by Gal4-UAS system. Concentration dependent 40 repetitions were conducted by two concentration range: 10^−5^–10^−2^ mg/ml range of standard LPS and 10^−1^–10 mg/ml range of standard LPS. (c) and (d) illustrated the grooming induction in control flies (siblings). Standard LPS was used as a stimulus. n = 40 (n = 20 for each sex). Data represents mean +/- SE, analysed as in Fig 2. A significant increase in response from that of the control (water) is indicated by asterisks: * indicates p < 0.05, ** indicates p < 0.01, and *** indicates p < 0.001 (Dunnett’s test).
(TIF)

S1 Table Results of Chi-square test on grooming induction by G+ microbe and highly purified microbial compounds in wild type flies. Microbes, which induced significant concentration-dependent behavioral increase were in white zone, and no-significance in gray zone. White zone in a table shows significant difference (p < 0.05), and grey zone indicates no-significance (p > 0.05).
(TIF)

S2 Table Results of Chi-square test on grooming induction in IMD pathway related mutants. Mutants, which showed significant concentration-dependent behavioral increase were in white zone, and no-significance in gray zone. White zone in a table shows significant difference (p < 0.05), and grey zone indicates no-significance (p > 0.05).
(TIF)

S3 Table Results of Chi square test on grooming induction in PGRP-LC mutants to quinine and in 10-day old PGRP-LC mutants. Mutants, which showed significant concentration-dependent behavioral increase were in white zone, and no-significance in gray zone. White zone in a table shows significant difference (p < 0.05), and grey zone indicates no-significance (p > 0.05).
(TIF)

S4 Table Results of Chi square test on grooming induction in PGRP-LC related mutants. Mutants, which showed significant concentration-dependent behavioral increase were in white zone, and no-significance in gray zone. White zone in a table shows significant difference (p < 0.05), and grey zone indicates no-significance (p > 0.05).
(TIF)
