# Supplementary figures and images for: The gram-negative sensing receptor PGRP-LC contributes to grooming induction in Drosophila
Source: PLoS One. 2017 Nov 9;12(11):e0185370. doi: 10.1371/journal.pone.0185370 (PMC5679552; doi:10.1371/journal.pone.0185370)

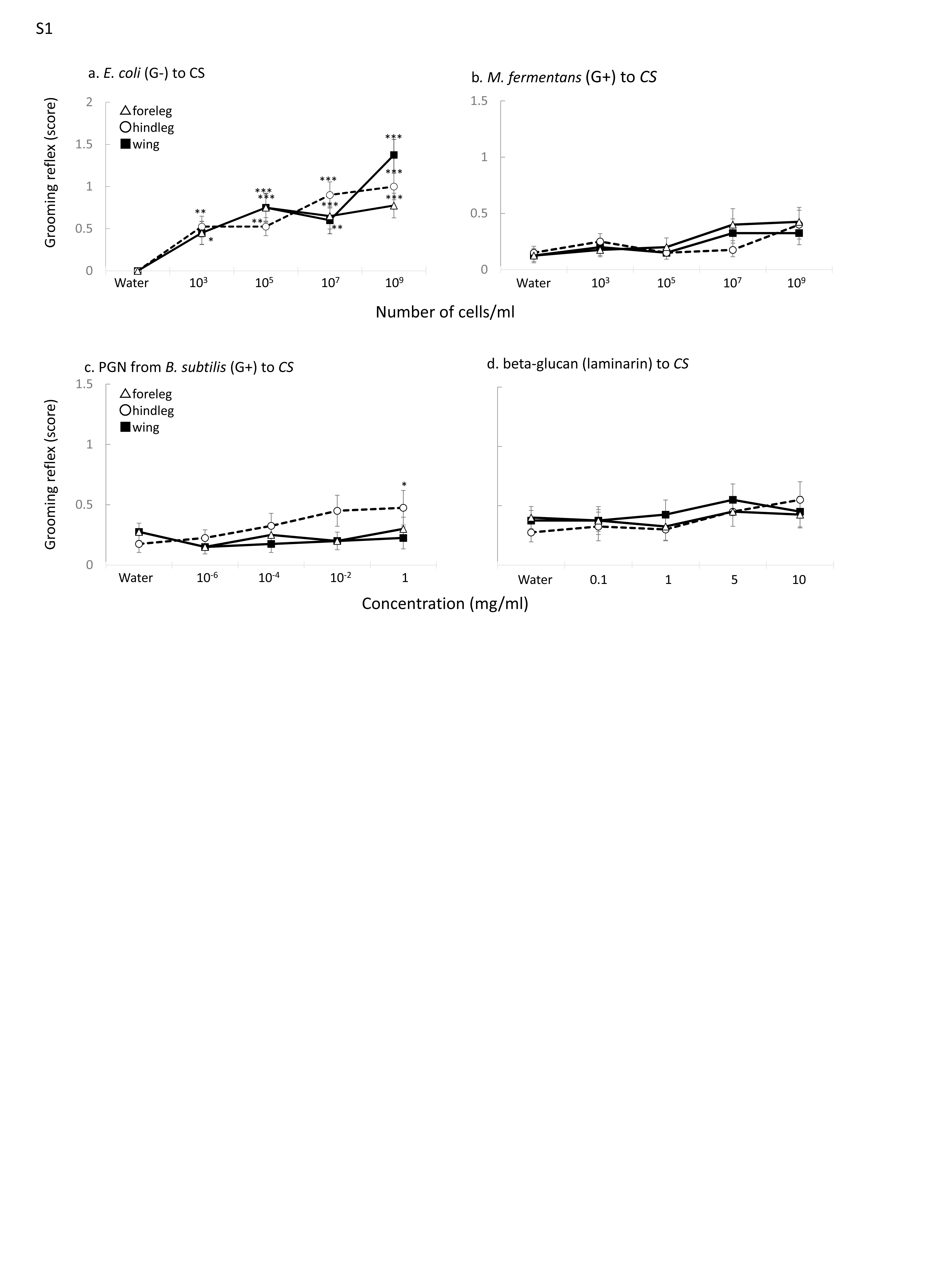

Supplement: S1 Fig — A significant increase in response from that of the control (water) is indicated by asterisks: * indicates p < 0.05, ** indicates p < 0.01, and *** indicates p < 0.001 (Dunnett’s test). Please note that the data of CS flies to E. coli is provided as a reference. This data belongs to the previous publication in Front. Behav. Neurosci. [5]. (TIF) [file pone.0185370.s001.tif]

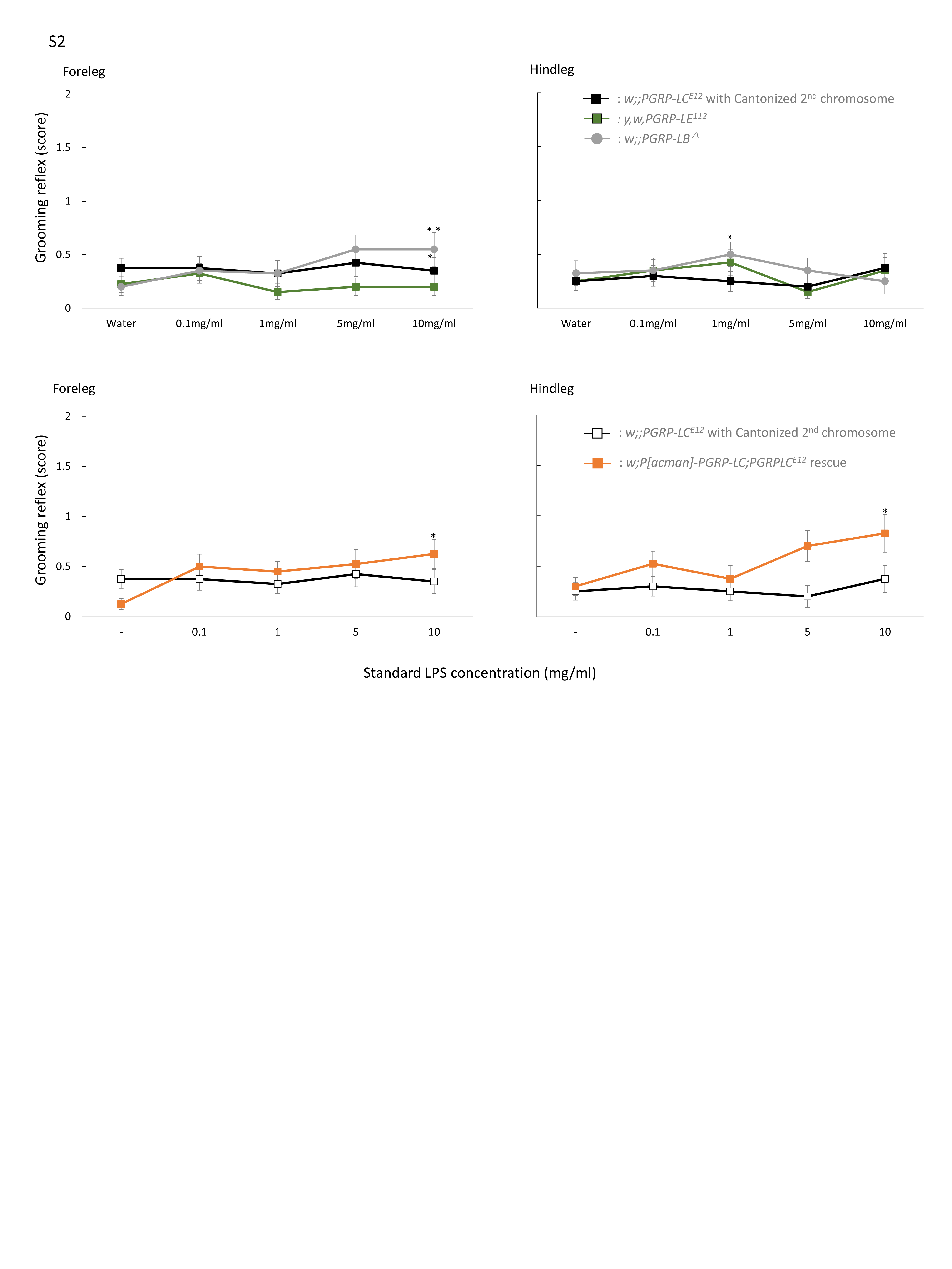

Supplement: S2 Fig — Standard LPS was used as a stimulus. n = 40 (n = 20 for each sex). Data represents mean +/- SE, analyzed as in Fig 2. A significant increase in response from that of the control (water) is indicated by asterisks: * indicates p < 0.05, ** indicates p < 0.01, and *** indicates p < 0.001 (Dunnett’s test). (TIF) [file pone.0185370.s002.tif]

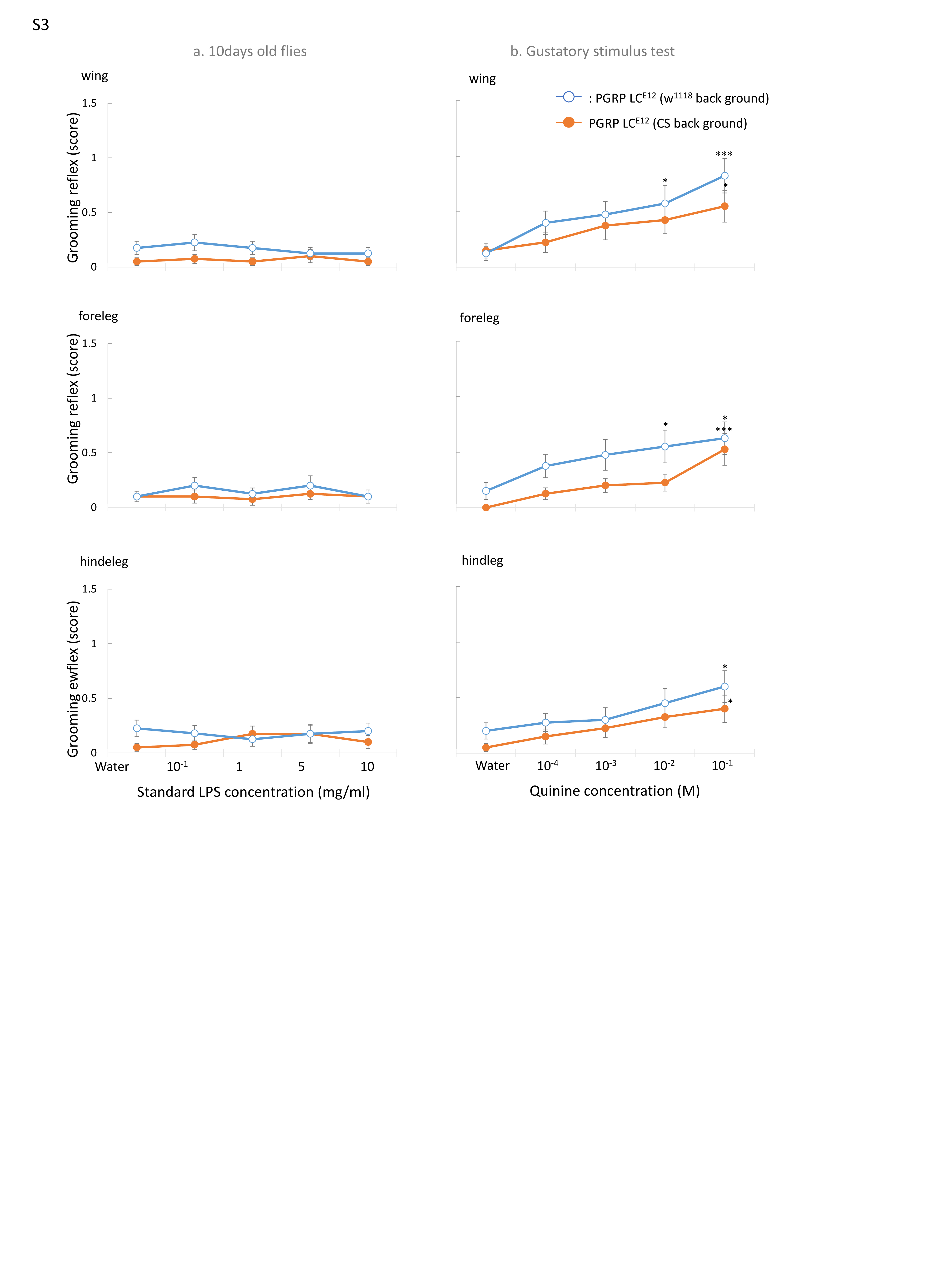

Supplement: S3 Fig — (a) Grooming behavior induced in 10-day old PGRP-LCE12 mutants in orange circle with orange line: CS background and white circle with blue line: w1118 background. (b) Grooming behavior induced by quinine in PGRP-LCE12 mutants of orange circle with orange line: CS background and white circle with blue line: w1118 background flies. Standard LPS was used as a stimulus. n = 40 (n = 20 for each sex). Data represents mean +/- SE, analysed as in Fig 2. A significant increase in response from that of the control (water) is indicated by asterisks: * indicates p < 0.05, ** indicates p < 0.01, and *** indicates p < 0.001 (Dunnett’s test). (TIF) [file pone.0185370.s003.tif]

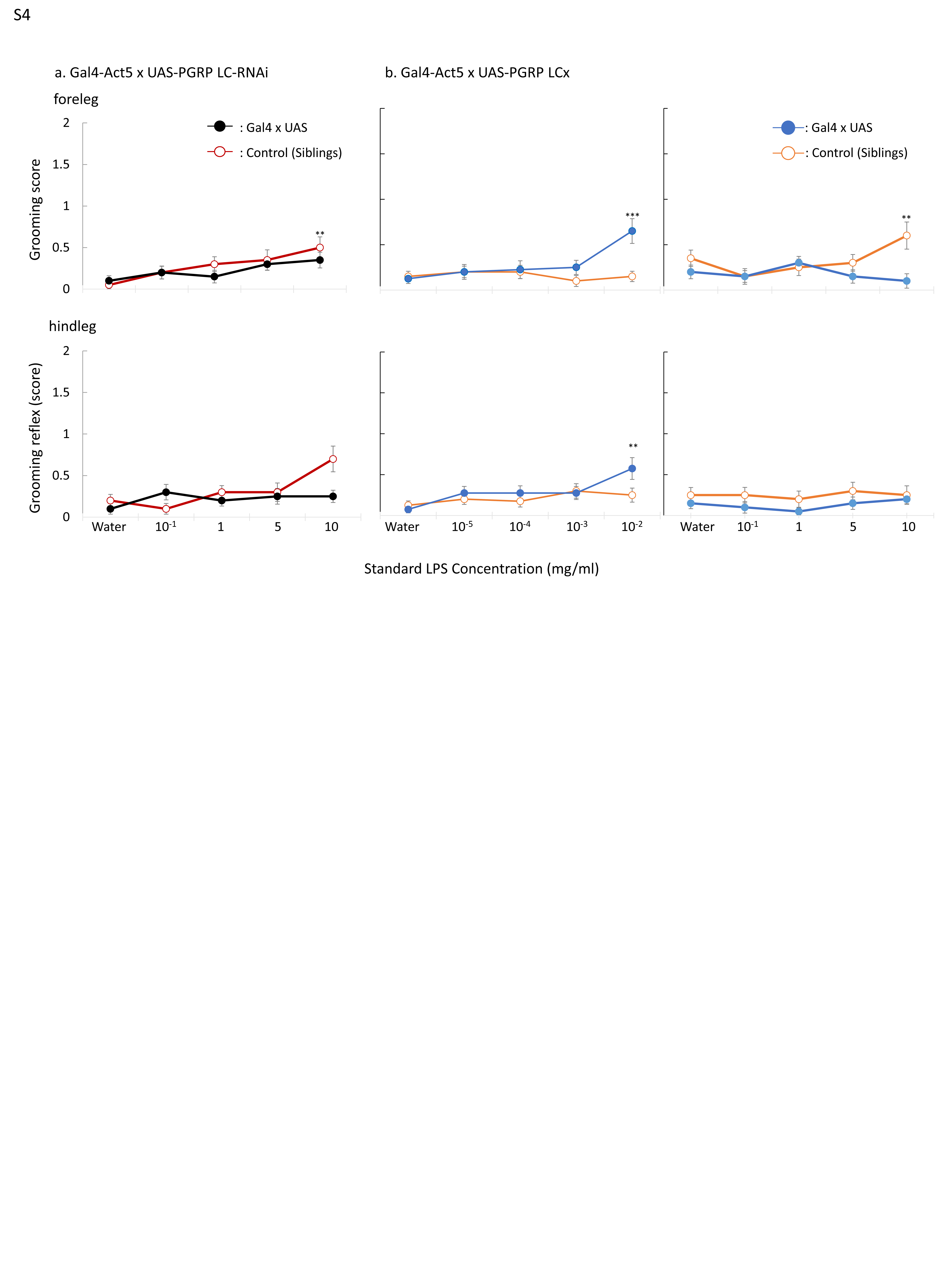

Supplement: S4 Fig — (a) Grooming behavior induced in offspring of act-Gal4 x PGRP-LCRNAi flies black circle with black line: actGal4x-PGRP-LCRNAi and white circle with red line control (siblings). (b) Grooming behavior induced by LPS contact on front/hind legs in PGRP-LCx overexpressing flies by Gal4-UAS system. Concentration dependent 40 repetitions were conducted by two concentration range: 10−5–10−2 mg/ml range of standard LPS and 10−1–10 mg/ml range of standard LPS. (c) and (d) illustrated the grooming induction in control flies (siblings). Standard LPS was used as a stimulus. n = 40 (n = 20 for each sex). Data represents mean +/- SE, analysed as in Fig 2. A significant increase in response from that of the control (water) is indicated by asterisks: * indicates p < 0.05, ** indicates p < 0.01, and *** indicates p < 0.001 (Dunnett’s test). (TIF) [file pone.0185370.s004.tif]

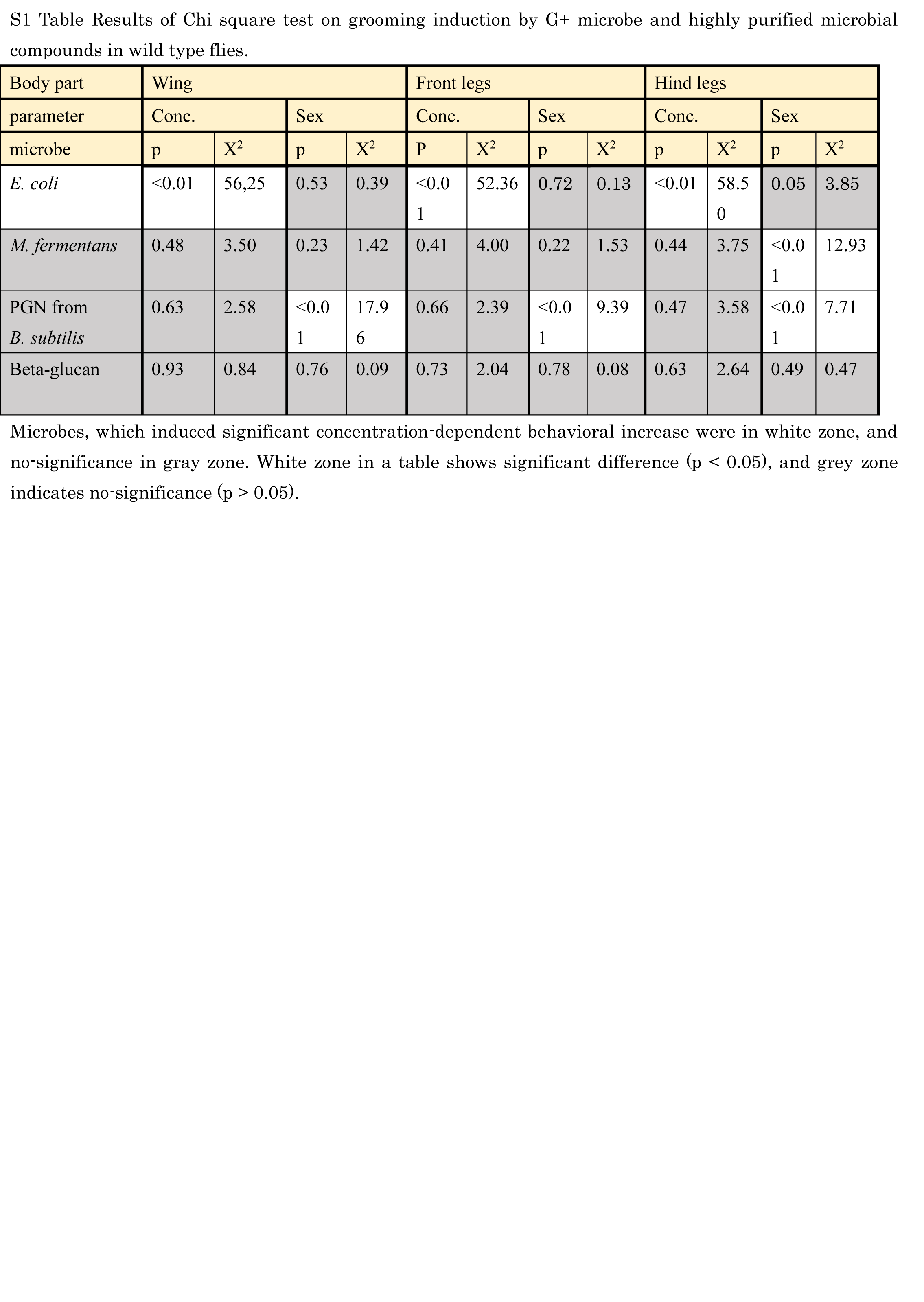

Supplement: S1 Table — Microbes, which induced significant concentration-dependent behavioral increase were in white zone, and no-significance in gray zone. White zone in a table shows significant difference (p < 0.05), and grey zone indicates no-significance (p > 0.05). (TIF) [file pone.0185370.s005.tif]

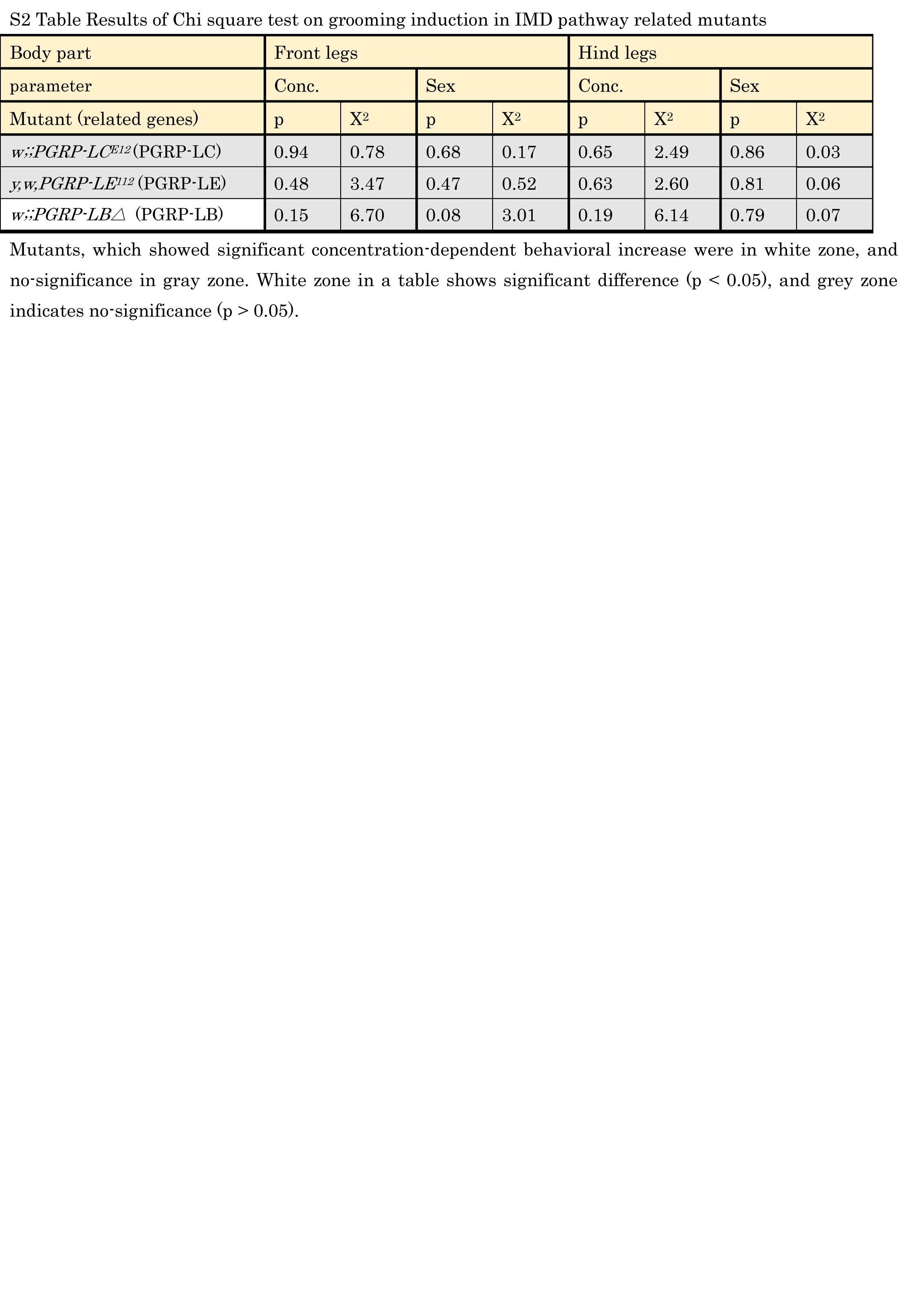

Supplement: S2 Table — Mutants, which showed significant concentration-dependent behavioral increase were in white zone, and no-significance in gray zone. White zone in a table shows significant difference (p < 0.05), and grey zone indicates no-significance (p > 0.05). (TIF) [file pone.0185370.s006.tif]

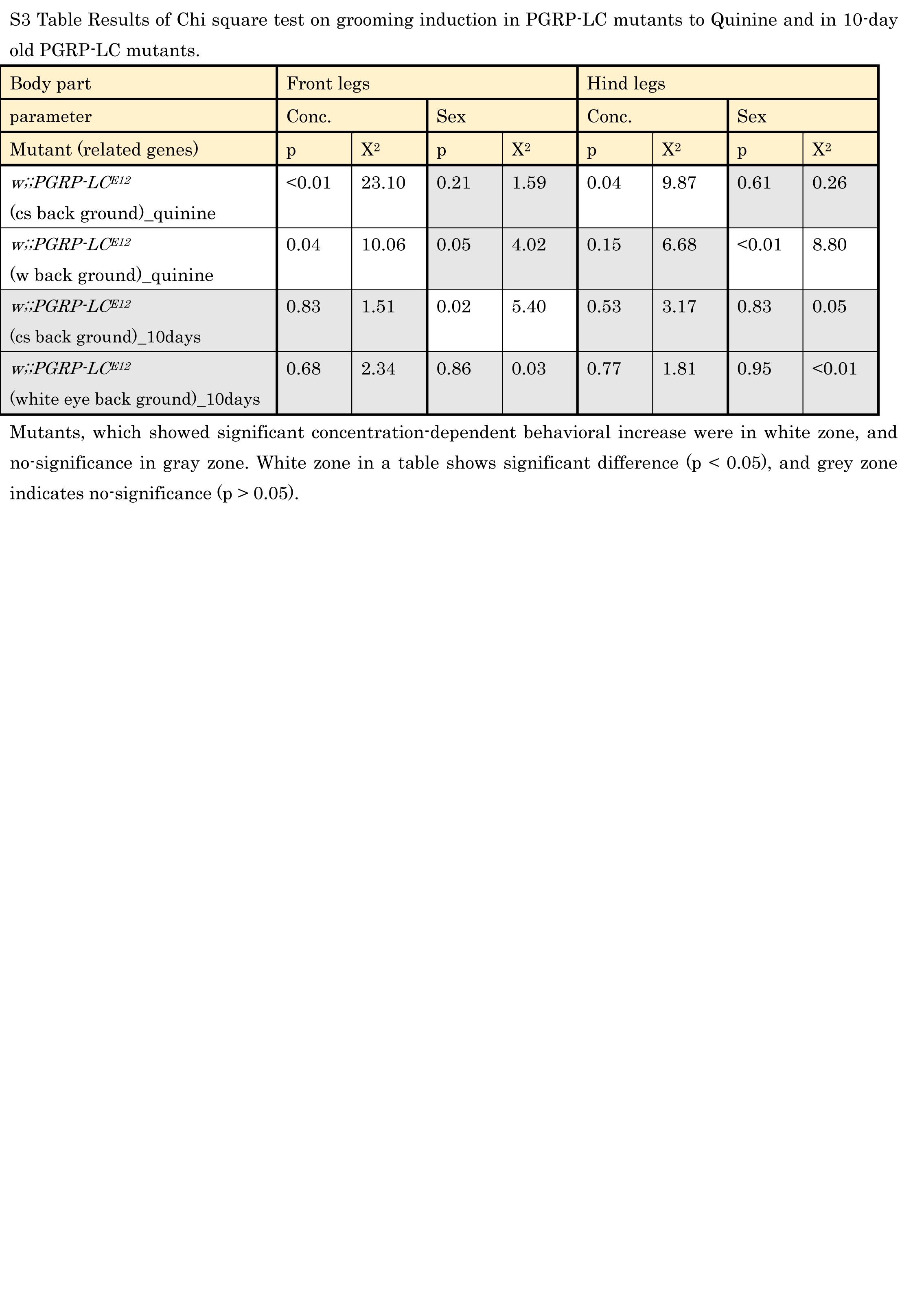

Supplement: S3 Table — Mutants, which showed significant concentration-dependent behavioral increase were in white zone, and no-significance in gray zone. White zone in a table shows significant difference (p < 0.05), and grey zone indicates no-significance (p > 0.05). (TIF) [file pone.0185370.s007.tif]

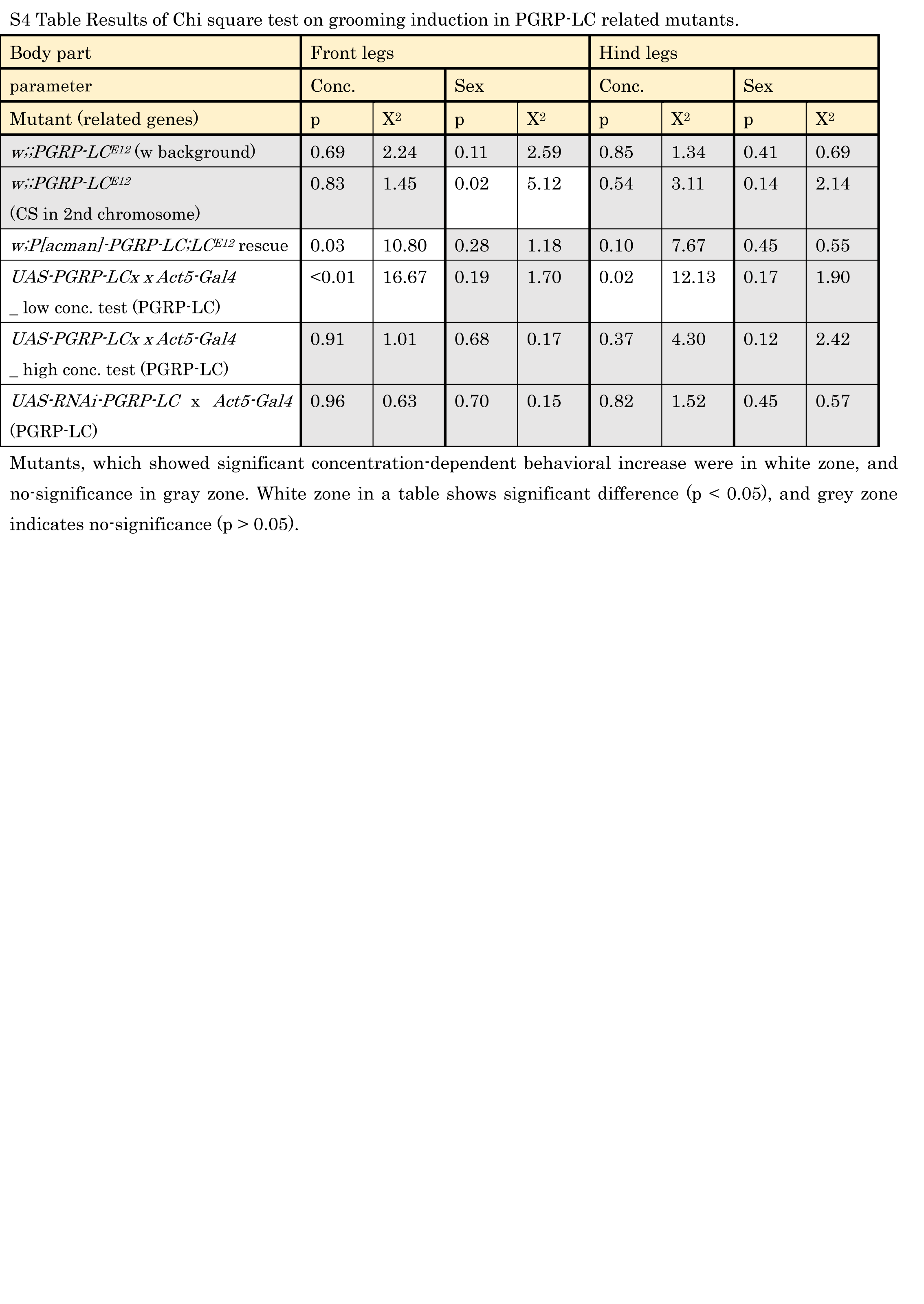

Supplement: S4 Table — Mutants, which showed significant concentration-dependent behavioral increase were in white zone, and no-significance in gray zone. White zone in a table shows significant difference (p < 0.05), and grey zone indicates no-significance (p > 0.05). (TIF) [file pone.0185370.s008.tif]
